# One-Step Electrical Insulating Oil Regeneration on Electret PVDF/BaTiO_3_ Composite Nanofibers

**DOI:** 10.3390/polym14132631

**Published:** 2022-06-28

**Authors:** Boyan Zhao, Yaxiong Tan, Feipeng Wang, Li Yang, Nuo Cheng

**Affiliations:** 1 State Key Lab of Power Transmission Equipment & System Security and New Technology, School of Electrical Engineering, Chongqing University, Chongqing 400044, China; 20196273@cqu.edu.cn (B.Z.); 20193453@cqu.edu.cn (N.C.); 2 CISDI Chongqing Information Technology Co., Ltd., Chongqing 400013, China; li.a.yang@cisdi.com.cn

**Keywords:** electret, insulating oil regeneration, PVDF/BaTiO_3_ nanofiber, electric field

## Abstract

Insulating oil is a pivotal component of power transformers, but it suffers from aging byproducts during service operation. The aging byproducts from the degradation of oil insulation tend to induce insulation failure, which poses a significant threat to the security of the power grid. Therefore, the regeneration of insulating oil is required to prolong the useful life of insulating oil and hence be of economic and ecological interests. Typical in-use oil regeneration routes employ multi-step procedures. In this work, a one-step regeneration method using a PVDF/BaTiO_3_ composite membrane is proposed. BaTiO_3_ endows the composite membrane with improved hydrophobicity and an electret state. The regeneration performance of the PVDF/BaTiO_3_ nanofiber membrane was assessed by considering the acid value, moisture content, dielectric loss factor tan δ, and the AC breakdown voltage of the refreshed oil. The test results showed that the filtration efficiencies toward formic acid and moisture were up to 77.5% and 60.6%, respectively. Moreover, the dielectric loss factor tan δ of the refreshed oil decreased evidently at a power frequency, and the AC breakdown voltage rose from 23.7 kV to 38.9 kV. This suggests that the PVDF/BaTiO_3_ composite membrane may be employed efficiently, and it minimizes aging byproducts via the one-step filtration.

## 1. Introduction

A power transformer is one of the key pieces of equipment in electrical substation systems [1,2,3,4]. As the key component of oil-immersed power transformers, insulating oil can extend the service life of power transformers by keeping the windings cool and suppressing arc generation [5,6,7]. Approximately 0.4 million tons of insulating oil are used worldwide in power equipment [8]. However, falling victim to the long-term impact of the thermal field and electromagnetic field, insulating oil is consistently undergoing oxidation that provokes byproducts such as carboxylic acid, ketone, aldehyde, alcohol, moisture, etc. [9,10] These byproducts elevate the acid value, moisture content, dielectric loss, and electrical conductivity of insulating oil, which can lead to permanent insulation failure and severe damage to power transformers and electrical substations.

The acidic substance may cause a significant dielectric loss of insulating oil, an increase in the corrosion rate of coils, and form sludges on the windings in power transformers. This reduces the heat transfer capability of the oil [11]. The decomposition products also attack the paper insulation of the transformer windings, and the paper loses its mechanical and dielectric properties [12]. Meanwhile, the increase in moisture content will predominantly reduce the breakdown voltage of insulating oil. High temperature cracking, aging, oxidative cracking, and hydrolysis of insulating paper within the power transformer promote the production of organic acids. Subsequently, these aging products will continue to accelerate the aging process. Instead of replacing insulating oil, the regeneration of insulating oil is a promising method that prolongs the transformer life and reduces carbon emissions [13,14,15]. Nevertheless, methods such as vacuum filtration, electrostatic filtration, and centrifugal separation require complex procedures and display relatively low efficiency in removing acidic byproducts, moisture, charged colloidal impurities, and sub-micron solid impurities. As a result, the regenerated insulating oil under such purification methods is not close to meeting the requirements of power transformers.

The morphology and membrane structure of the filtration membrane is closely associated with the efficiency of the regeneration process. Nanofiber membranes have demonstrated high filtration efficiency due to their smaller pore size. Nevertheless, filtering membranes are frequently challenged by acidic corruption and liquid shocks from flowing insulating oil during aged oil regeneration. PVDF exhibits exceptional electret performance as well as excellent chemical stability. PVDF also possesses excellent ductility. In addition to the mechanical blocking impact of an electret within the nanofiber membrane, the membrane can effectively improve the filtration accuracy by confining small-sized impurities via the electrostatic influences between the nanofibers. The corona charging process can accomplish an in situ polarization of the nanofiber, while the electric charges stay stabilized for a fairly long time [16,17,18]. Hence, PVDF nanofiber membranes fabricated from electro-spinning can potentially be used in air purification [16].

Yang et al. conducted dielectric tests on filtered aged insulating oil with PVDF nanofiber membranes fabricated via electrospinning. After filtration, the AC breakdown and dielectric loss factor at the power frequency of the insulating oil were evidently improved. Additionally, the acid value of the filtered insulating oil was significantly reduced.

To further expand the filtration efficiency, nanofiber membranes with different barium titanate (BaTiO_3_) nanoparticle doping concentrations were prepared. The impacts caused by the BaTiO_3_ doping concentration on the nanofiber membrane’s surface morphology, charge stability, and hydrophobicity were studied. Through comparisons involving the acid value, moisture content, dielectric properties, and the AC breakdown voltage of insulating oil, the performance of the PVDF/BaTiO_3_ composite membrane for insulating oil regeneration was analyzed [19].

## 2. Materials and Methods

### 2.1. Material

The PVDF powder (FR904, Mw = 680,000) was supplied by Shanghai 3F, China. The N-dimethyl formamide (DMF) with a purity better than 99.5% was considered as a solvent and was purchased from Chengdu Chron Chemicals, China. They were both used as received without further purification. Polypropylene (PP) nonwoven membranes of 250 μm in thickness with a weight density of 30 g/m^2^ were provided by Zhejiang Zhaohui, China. The 40 nm barium titanium trioxide (BaTiO_3_) nanoparticles with a purity better than 99.99% were provided by Aladdin Chemicals, China.

### 2.2. Nanofiber Preparation

In order to optimize the electrospinning parameters, the PVDF in a DMF solution with a concentration of 12 wt% was prepared. The BaTiO_3_ was dispersed first in DMF by sonication, and then both solutes, BaTiO_3_ and PVDF, were stirred further by adding the proper amount of PVDF to the solution to achieve the weight ratio of BaTiO_3_/PVDF, varying from 0 to 15 wt% by steps of 2.5 wt%. All the prepared solutions were subjected to degassing in vacuum at room temperature (ca. 25 °C).

**Figure 1:**
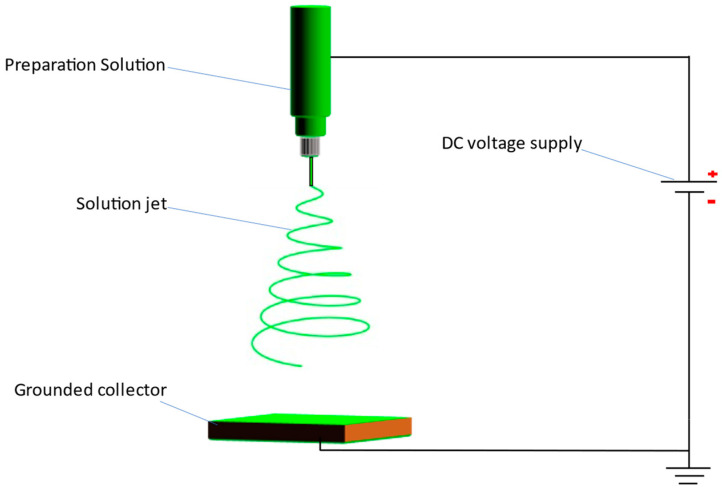
The setup for electrospinning.

The setup for electrospinning is shown in Figure 1. The electrospinning was performed at 35 °C and a relative humidity of 35%. The distance between the stainless-steel needle tip (radius of 0.8 mm) and the grounded collector was 20 cm. The voltage on the tip was set to 20 kV by a DC power supply, while a digital syringe pump was used to control the propulsive rate. The fabrication process during electrospinning lasted about 3 h and differed due to the variation in BaTiO_3_ contents in the different test groups. The higher the BaTiO_3_ content, the longer the time needed for electrospinning.

### 2.3. Characterizations

The morphology of the nanofibers was observed by a field-emission scanning electron microscope (FESEM, MIR3TM, Tescan, Brno, Czech). The SEM was equipped with a high-accuracy energy-dispersive spectroscope (EDS, GENESIS XM, EDAX, Osaka, Japan), which was feasible to examine the distribution of nanoparticles within the PVDF matrix. The hydrophobicity of the nanofibers was quantified by measuring the water contact angles.

**Figure 2:**
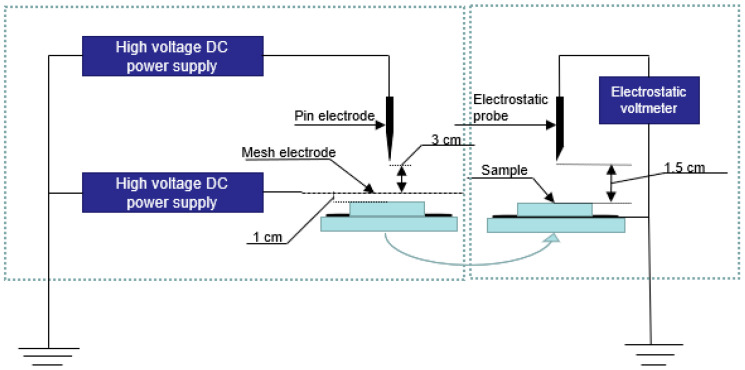
Setup for corona charging polarization and surface potential measurement system.

In order to identify the charge stability of the nanofibers, corona charging was performed by a tri-electrode system, as in Figure 2. During the 3 min corona charging, the voltages at the needle and the grid were set to 7 and 2 kV, respectively. An electrostatic voltmeter (542A, Trek, Lockport, NY, USA) was utilized to monitor the surface potential decay (SPD) of the samples at 90 °C.

A sand core funnel equipped with a vacuum pump was employed to examine the oil regeneration performance of the nanofibers via measurements of the moisture, acid value, dielectric dissipation factor, resistance, and breakdown voltage. To testify the validity of the filtration performance tests, at least six circle-shaped membrane samples with diameters of 2 cm were applied in each set of experiments.

During the preparation procedure of the formic acid test, the Karamay 25# mineral oil was first dried at 50 °C at 50~100 Pa for 48 h. For every 2 g of mineral oil, 1 μL of formic acid was added. The resultant was then shaken in an ultrasonic cleaner for 30 min, to achieve homogeneous dispersion. The acid value of the manufactured mineral oil was 0.551 mg KOH/g.

## 3. Results and Discussion

### 3.1. Morphology of Nanofibers

**Figure 3:**
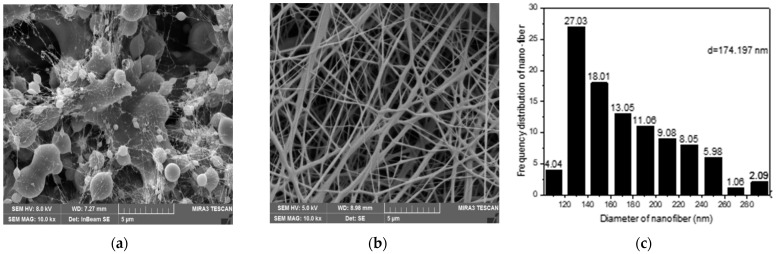
SEM images of nanofibers spun from PVDF solution with concentrations of (**a**) 6 wt% and (**b**) 12 wt%. The diameter distribution of nanofibers in (**b**) is shown in (**c**).

Figure 3a,b show the scanning electron microscope (SEM) images spun from PVDF solutions with either 6% or 12% concentrations. There are numerous bead strings in Figure 3a, which are likely due to the low solution concentration. Upon such a case, the “liquid fibers” were not able to be sufficiently solidified while flying from the energized tip to the grounded collector, which should result in the so-called “wet fibers”. Because of the surface tension force, the “wet-fibers” tended to shrink and to form a ball-shaped morphology, i.e., the resultant bead strings in Figure 3a. The higher PVDF concentration turned the situation from numerous to none, as evidenced by the well-formed fibers in Figure 3b. It is indicated that the fibers, upon a proper concentration, e.g., 12%, are feasible to obtain “dry fibers” on the collector. An image-analyzing program was used to quantify the diameter distribution of the nanofibers, which is shown in Figure 3b. Figure 3c provides the diameter-dependent frequency distribution of the nanofibers. It is seen that more than 50% of nanofibers had diameters ranging from 130 to 170 nm, which indicates a well-narrowed distribution and is expected to realize controllable fabrication.

The majority of fiber diameters were in the range between 130 and 180 nm, with an average size of 150 nm. The coefficient value (C.V.) of the fiber diameter was kept at merely 0.25, suggesting that fiber diameter forms a homogenous distribution. In order to obtain membranes with expectable insulating oil regeneration properties, it can be concluded that a 12 wt% PVDF fabrication membrane is the most suitable condition.

**Figure 4:**
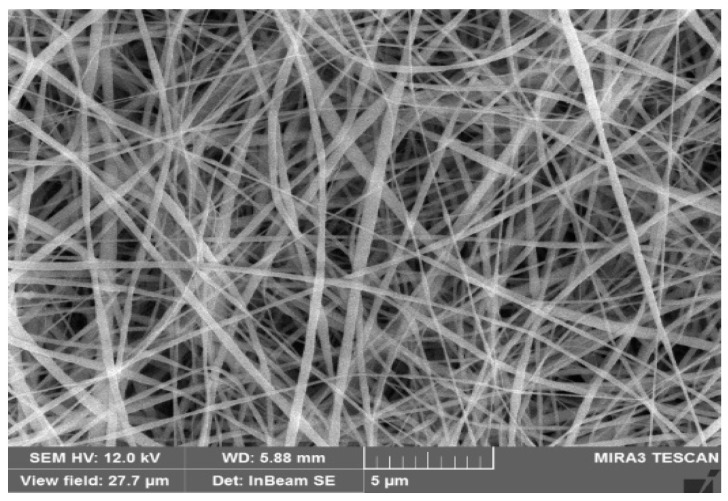
SEM graph of nanofiber from group 10 wt% BaTiO_3_ concentration in PVDF.

In order to recognize the influence brought by the addition of BaTiO_3_, SEM analysis techniques identical to those applied in Figure 3 were utilized for Figure 4 as well. The results are shown in Table 1 and Figure 4.

Figure 4 and Table 1 listed a number of significant diameters of the nanofibers fabricated after the addition of BaTiO_3_ in the PVDF electro-spinning preparation solution. As BaTiO_3_ content increased, the average nanofiber diameter displayed a tendency to rise when BaTiO_3_ content was under 10 wt% and gradually dropped when BaTiO_3_ was higher. When the BaTiO_3_ concentration was kept at 10 wt%, the nanofibers reached a series of dimensions with an average pore size of 0.81 μm and an average fiber diameter of 174 nm.

Furthermore, the average nanofiber diameter from the group with 10 wt% BaTiO_3_ reached a homogenous distribution where the C.V. was narrowed down to ca. 0.26. These fiber dimensions are considered the most suitable for fabricating an effective membrane.

Finally, typical nanofibers with a proper concentration of 10 wt% are shown in the following SEM in Figure 4. It is recognized that the addition of 10 wt% BaTiO_3_ did not disturb the characteristics of the fiber condition from pure 12 wt% PVDF. Therefore, given all the fiber dimensions, it is safe to conclude that nanofibers prepared under a 10 wt% BaTiO_3_ concentration were in excellent condition.

### 3.2. Charge Stability of PVDF/BaTiO_3_ Nanofibers

Considering the intrinsic polar property of PVDF and BaTiO_3_ and the possible poling during electro-spinning, the obtained composite nanofibers were expected to contain both space charges and oriented dipole charges. Space charges are introduced during electro-spinning, and dipole charges are possibly formed due to the electric field generated by space charges. Therefore, we used surface potential decay (SPD) to identify the charge stability of the composite nanofibers.

**Figure 5:**
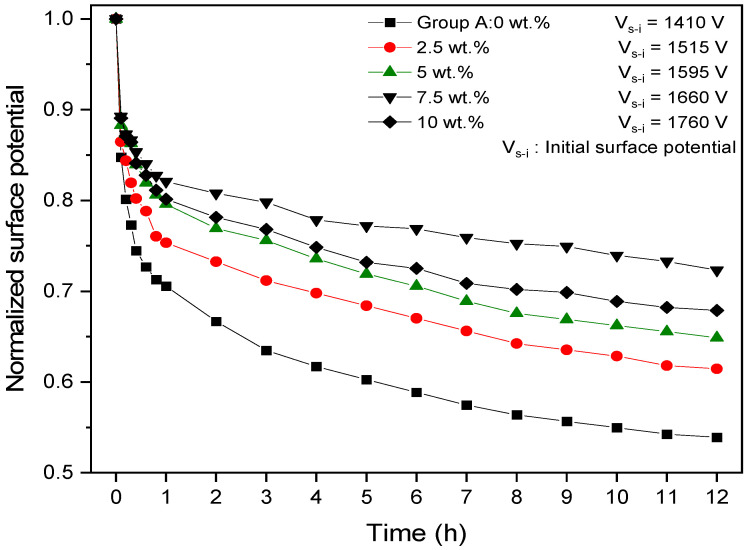
Surface potential decay of PVDF/BaTiO_3_ nanofiber composite membranes.

Figure 5 includes the surface potential decay curves of composite nanofibers with BaTiO_3_ contents varying from 0 to 10 wt%. The surface potentials (SPs) of nanofibers, regardless of BaTiO_3_ content, all demonstrated relatively fast paced decay within the first 2 h. Subsequently, during the latter 10 h, the nanofibers with different BaTiO_3_ contents began to show evident differences in the SPD rates. Distinctive from pure PVDF fiber, the SPs of nanofibers, regardless of their BaTiO_3_ contents, all displayed reduced decay rates that were evidenced by low SP value drops during the same time period, indicating that the addition of BaTiO_3_ was beneficial to the improvement of nanofiber charge stability. When the concentration of BaTiO_3_ was higher or lower than 7.5 wt%, the decay rate of the surface potential was inversely or directly proportional to the doping concentration of BaTiO_3_, respectively.

The surface initial voltages (Vs-i) of different samples are listed in the upper right corner of Figure 5. The V_s-i_ values increases with increasing BaTiO_3_ doping concentrations and reached a maximum of 1760 V with a doping concentration of 10 wt%. Since the Vs-i value remained at a higher degree during the same amount of time, there is reason to believe that the addition of BaTiO_3_ creates considerable interference, which is beneficial to the formation of more energy traps. Subsequently, excellent charge stability is achieved.

The decay of surface potentials for samples with 7.5 wt% and 10 wt% concentrations were comparatively slower than the other samples. Nevertheless, in the case where fibers shared the same thickness, larger surface potentials guaranteed a stronger and more stabilized electric field. Given that 10 wt% possessed the highest initial surface potential voltage, we can conclude that the optimal concentration of BaTiO_3_ for nanofiber charge stability is 10 wt%.

### 3.3. Hydrophobicity of PVDF/BaTiO_3_ Nanofiber Membrane

As moisture increases, the enhanced space charge accumulation and electric field distortion may pose a threat to the insulating properties of oil-paper insulation [20]. Therefore, the moisture content within the insulating oil should be confined. It is universally acknowledged that moisture removal is closely associated with the hydrophobicity of the filtration surface. Therefore, in order to achieve high moisture removal efficiency with membranes made out of PVDF/BaTiO_3_ nanofibers, their hydrophobicity ought to be probed.

**Figure 6:**
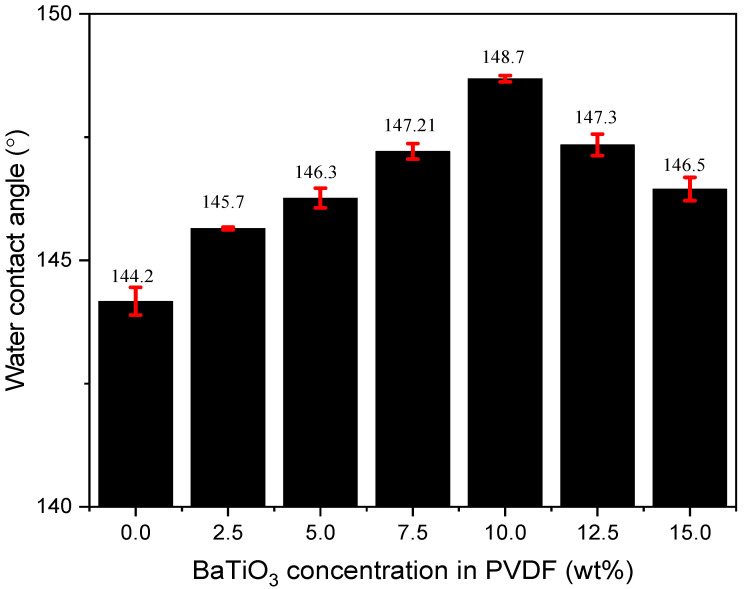
Water contact angle of PVDF/BaTiO_3_ nanofiber composite membranes.

The water contact angles (WCA) of nanofibers with different BaTiO_3_ concentrations were calculated by an optical contact angle measuring instrument, as previously mentioned in Section 2.3. The relationship between the WCA and the BaTiO_3_ doping concentration is shown in Figure 6:

The water contact angle first increased with increasing doping concentration and eventually reached a maximum value of 149° at 10 wt%. The WCA started to decrease with further increases in the BaTiO_3_ doping concentration. The addition of BaTiO_3_ increases the roughness of PVDF fibers and the air content within the grooves between rough fiber surfaces. Subsequently, the contact area between water and the membrane surface is reduced, as the contact area between air and the membrane surface is increased. Subsequently, the WCA of PVDF/BaTiO_3_ nanofibers is increased.

Such nanofibers with excellent hydrophobicity could be used in the process of high-efficiency insulating oil regeneration. When flowing insulating oil is subjected to PVDF/BaTiO_3_ composite membranes, it would be particularly difficult for moisture to penetrate through the layers within the membrane thanks to the high WCA. Therefore, it is possible for moisture to be efficiently removed from flowing insulating oil.

## 4. Evaluation of Insulating Oil Regeneration Performance

### 4.1. Regeneration of Insulating Oil

A. Performance on Formic Acid Filtration

**Figure 7:**
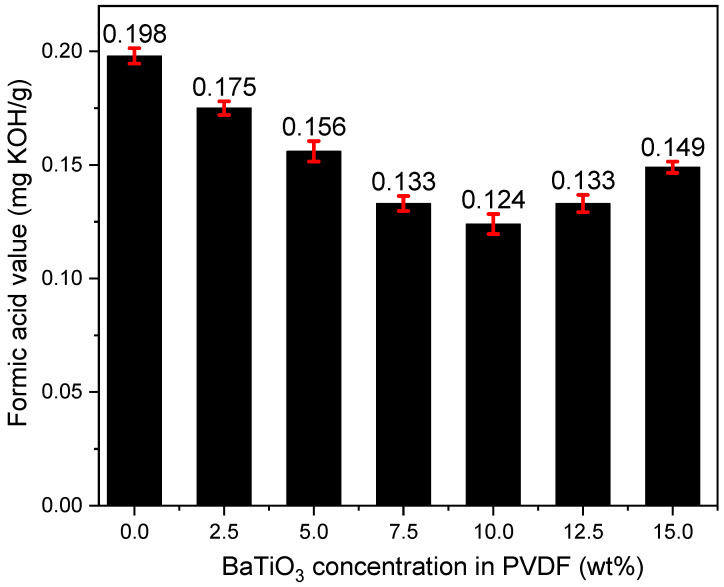
Acid values after filtering formic acid mineral oil mixture with PVDF/BaTiO_3_ nanofiber composite membranes. Formic acid value before filtration was 0.551 mg KOH/g.

The mixture of various acids within the oil can be traced back to hydrolysis and a carbonate chain reaction during the aging process of insulating oil. Nevertheless, there is hardly any chance for an accurate replica covering the infinite possibilities of aging insulating. Therefore, in order to tap the acid removal potential of PVDF/BaTiO_3_ composite membranes, formic acid was chosen as a substitute for acidic byproducts since it holds the smallest particle dimension among all acids. Insulating oil with formic acid was prepared as mentioned in 2.3. The acid values of differing test groups are listed in Figure 7.

The removal efficiency of formic acid increased with the increase in the BaTiO_3_ concentration. The acid value of the mineral oil mixture after filtration was inversely proportional to the doping concentration of BaTiO_3_, reaching a minimum of 0.124 mg KOH/g at 10 wt%.

As mentioned in Section 2.3., the initial acid value of prepared insulating oil is 0.551 mg/L. Given the acid value measured after a one-time regeneration with a 10 wt% PVDF/BaTiO_3_ composite membrane, the calculated results show that the formic acid removal efficiency under such a condition approached 77.5%, which is the highest value among all the samples. Once the doping concentration exceeded 10 wt%, the acid value tended to rise as BaTiO_3_ doping concentration increased, which means that formic acid removal efficiency was lowered.

The high acid filtration efficiency of 10 wt% BaTiO_3_ membrane is evidenced by the lowest acid value shown in Figure 7, suggesting that even formic acid can be smoothly removed with merely a one-time regeneration. Such progress is likely attributed to the homogenously distributed fiber diameters and the pore size of PVDF/BaTiO_3_ membranes.

B. Acid Value after Filtration

**Figure 8:**
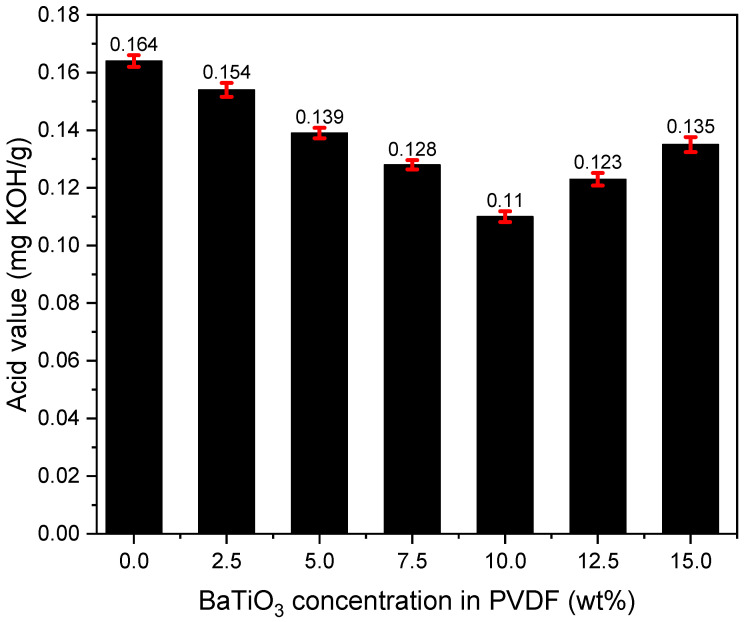
Acid value after filtering insulating oil with PVDF/BaTiO_3_ nanofiber composite membrane. Acid value before filtration was 0.241 mg KOH/g.

After comparing all test results from Figure 8 with the original acid value before oil regeneration (0.241 mg KOH/g), there is reason to believe that PVDF membranes of different doping concentrations all possess the ability to reduce the acid content of aged insulating oil, supporting the theory that PVDF/BaTiO_3_ membranes hold the potential to filter all sorts of byproduct acids. Additionally, the excellent filtration behavior of the 10% doping concentration membrane was spotted. The acid value of the insulating oil after the first-time filtration was 0.11 mg KOH/g, and the filtration efficiency was 48.6%, which was 25.2% more efficient than the conventional PVDF membrane.

Since acids other than formic acid possess larger sizes on the molecule level, it is harder for acids to penetrate through pores on the filtration membrane. Thus, the common acid filtration efficiency of the PVDF/BaTiO_3_ composite membrane after a one-time regeneration is expected to be higher than 77.5%, which was the removal efficiency of formic acid.

Based on our results, we concluded that external particles in the aging insulating oil not only involve acidic colloid but also solid particles, most of which are larger than formic acid. Therefore, it is reasonable to speculate that the removal efficiency of our regeneration method towards acidic byproducts could be even higher than 77.5%.

C. Moisture content within insulating oil

The moisture content after oil regeneration acts as a rule to study the removal efficiency of moisture. Figure 8 includes the moisture content in the insulating oil after regeneration with composite nanofibers with BaTiO_3_ contents varying from 0 to 10 wt%.

**Figure 9:**
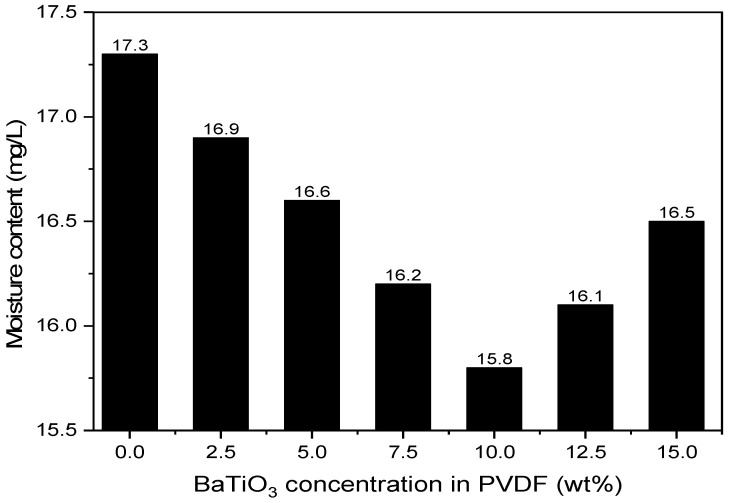
Moisture content of insulating oil after membrane filtration. Moisture content before filtration was 40.1 mg/L.

Given that the actual moisture content within aging insulating oil is 40.1 mg/L, the test results from Figure 9 pointed out that the PVDF/BaTiO_3_ membrane commits a great contribution to the significant drop in moisture content. The moisture content within insulating oil reached a minimum of 15.8 mg/L after the regeneration with 10 wt% concentration, displaying a filtration efficiency at 60.6% and elevating the filtration efficiency by 3.2%. The quality of the insulating oil after the first-time filtration was merely 5% away from meeting the general standards.

D. Dielectric loss factor after filtration

The actual dielectric loss factor tan δ of aged insulating oil is 1.439% at 90 °C and 50 Hz. By comparison, the dielectric loss factor tan δ of insulating oil after the regeneration of nanofiber membranes with varying BaTiO_3_ concentrations from 0 to 15 wt% all displayed obvious decreasing in dielectric factor tan δ. When the BaTiO_3_ concentration within PVDF was kept at 10 wt%, dielectric loss factor tan δ was lowered to 0.958%, improving the dielectric loss factor by 33.4% and 19.9%, compared to aged insulating oil before and after filtration by a pure PVDF membrane, respectively. This quality of insulating oil outperformed the general standard, which is tan δ ≤ 2%, to be specific.

**Figure 10:**
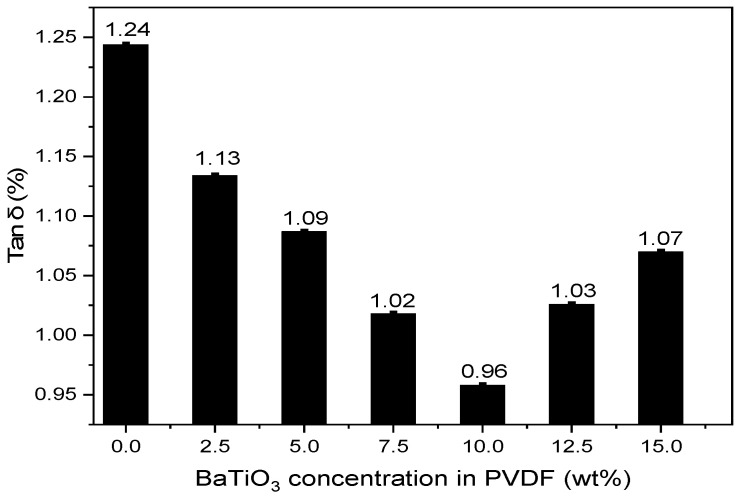
Dielectric loss factor (tan δ) after filtering insulating oil with PVDF/BaTiO_3_ nanofiber composite membranes.

As is known to all, the dielectric loss factor is closely associated with conducting loss and polarization loss within the oil. More external particles contained in aged insulating oil will lead to a higher conductive carrier concentration and an enhanced conductive rate. Subsequently, a high conductivity leads to a high dielectric loss. Therefore, it can be concluded that the low dielectric loss observed in Figure 10 suggests that the PVDF/BaTiO_3_ membrane filtration efficiency on external particles is satisfactory.

E. Breakdown Voltage after filtration

**Figure 11:**
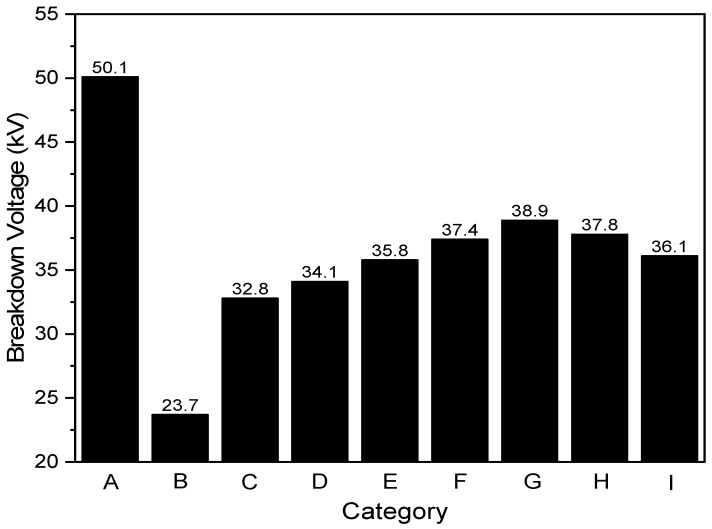
Breakdown voltage of insulating oil after one-time filtration with PVDF/BaTiO_3_ nanofiber composite membranes. The breakdown voltage before filtration was 23.7 kV.

Table 2 listed the sample oils for breakdown voltage test. The average initial breakdown voltages of samples A B were 23.7 kV and 50.1 kV, respectively, as shown in Figure 11. Samples D to I displayed higher breakdown voltages than those of samples B and C. Compared with sample B, sample G demonstrated the highest breakdown voltage of 38.9 kV, increasing it by 64.1%, while sample C merely elevated the breakdown voltage by 25.7%. This suggests that one-time filtration with PVDF/BaTiO_3_ composite membranes partially recovered the insulating properties within the oil and possessed a higher filtration efficiency than one-time filtration with a pure PVDF membrane. This improvement outperformed the general standard for the relevant breakdown voltage (30 kV). Overall, it is reasonable to conclude that a one-step regeneration with PVDF/BaTiO_3_ nanofibers is feasible to achieve higher efficiency insulating oil regeneration.

### 4.2. Mechanism of High-Efficiency Regeneration of Insulating Oil

It is obvious to say that nanofibers have a significant advantage in removing fine particles from a fluid due to their excellent mechanical blocking effect, which has been evidenced by numerous studies. Thanks to the high porosity observed in the high-definition SEM figures (e.g., Figure 3b), the improved mechanical blocking does not bring a pressure drop increase during filtration. In addition, the hydrophobicity of the PVDF/BaTiO_3_ composite nanofibers is raised by involving BaTiO_3_, and it plays a significant effect on removing moisture from insulating oil. This should be sourced to increase the fiber-roughness of BaTiO_3_ despite its relatively high hydrophilicity. Further, the addition of BaTiO_3_ tends to enhance the electric performance of PVDF, which should reasonably intensify the local electric field around the nanofibers and therefore endows the possibility to polarize the aging byproducts. This is justified by the distinctive acid removal capability, particularly the performance of clearing formic acid, which is the smallest organic acid, in insulating oil (cf. Figure 7). With regard to the acidic aging byproducts in insulating oil, typically large-sized acid molecules such as acetic acid and furfural, the nanofibers present with a higher capacity for acid removal (cf. Figure 8), which provides evidence on a microscopic level. Last but not least, the testing results from the circle-shaped membrane samples mentioned in Section 2.3. proved that our composite membrane samples can process 1500–2000 mL of insulation oil regeneration. This suggests that our membranes hold great potential in commercialization.

## 5. Conclusions

The PVDF/BaTiO_3_ composite nanofibers are fabricated by optimized electro-spinning parameters. The addition of BaTiO_3_ into PVDF improves hydrophobicity and electrical properties. The regeneration was able to lower the acid value of aged insulating oil from 0.241 to 0.11 mg KOH/g. Further, the moisture in the oil dropped from 17.3 to 15.8 mg/L, and the breakdown voltage recovered by 64.14% (i.e., from 23.7 to 38.9 kV). The PVDF/BaTiO_3_ composite nanofibers are potential candidates to regenerate aged insulating oil.

## Figures and Tables

**Table 1:** Main diameter and discrete coefficient of PVDF/BaTiO3 nanofiber composite membranes.

BaTiO_3_ Concentration within PVDF (wt%)	Minimum Pore Size (μm)	Maximum Pore Size (μm)	Average Pore Size (μm)	Average Fiber Diameter (nm)	C.V. Efficient
0	0.49	0.84	0.57	98	0.25
2.5	0.86	1.37	1.04	200	0.20
5	0.88	1.40	1.01	189	0.28
7.5	0.79	1.31	0.94	167	0.27
10	0.71	1.14	0.81	174	0.26
12.5	0.89	1.68	1.11	155	0.33
15	0.94	1.71	1.18	141	0.36

**Table 2:** Sample oils for breakdown voltage test.

Sample Oil	Oil Info
A	pure insulating oil
B	insulating oil before filtration
C	pure PVDF membrane
D	PVDF and 2.5 wt% BaTiO_3_ composite membrane
E	PVDF and 5.0 wt% BaTiO_3_ composite membrane
F	PVDF and 7.5 wt% BaTiO_3_ composite membrane
G	PVDF and 10 wt% BaTiO_3_ composite membrane
H	PVDF and 12.5 wt% BaTiO_3_ composite membrane
I	PVDF and 15 wt% BaTiO_3_ composite membrane

## Data Availability

The data that support the findings of this study are available from the corresponding author upon reasonable request.
